# Effect of nanoparticles on red clover and its symbiotic microorganisms

**DOI:** 10.1186/s12951-016-0188-7

**Published:** 2016-05-10

**Authors:** Janine Moll, Alexander Gogos, Thomas D. Bucheli, Franco Widmer, Marcel G. A. van der Heijden

**Affiliations:** Agroscope, Institute for Sustainability Sciences ISS, 8046 Zurich, Switzerland; Plant-Microbe-Interactions, Department of Biology, Utrecht University, 3508 TB Utrecht, The Netherlands; Institute of Evolutionary Biology and Environmental Studies, University of Zurich, Winterthurerstrasse 190, 8057 Zurich, Switzerland

**Keywords:** Nanomaterials, Agriculture, Crop, Beneficial soil microbes, Ecosystem services

## Abstract

**Background:**

Nanoparticles are produced and used worldwide and are released to the environment, e.g., into soil systems. Titanium dioxide (TiO_2_) nanoparticles (NPs), carbon nanotubes (CNTs) and cerium dioxide (CeO_2_) NPs are among the ten most produced NPs and it is therefore important to test, whether these NPs affect plants and symbiotic microorganisms that help plants to acquire nutrients. In this part of a joint companion study, we spiked an agricultural soil with TiO_2_ NPs, multi walled CNTs (MWCNTs), and CeO_2_ NPs and we examined effects of these NP on red clover, biological nitrogen fixation by rhizobia and on root colonization of arbuscular mycorrhizal fungi (AMF). We also tested whether effects depended on the concentrations of the applied NPs.

**Results:**

Plant biomass and AMF root colonization were not negatively affected by NP exposure. The number of flowers was statistically lower in pots treated with 3 mg kg^−1^ MWCNT, and nitrogen fixation slightly increased at 3000 mg kg^−1^ MWCNT.

**Conclusions:**

This study revealed that red clover was more sensitive to MWCNTs than TiO_2_ and CeO_2_ NPs. Further studies are necessary for finding general patterns and investigating mechanisms behind the effects of NPs on plants and plant symbionts.

**Electronic supplementary material:**

The online version of this article (doi:10.1186/s12951-016-0188-7) contains supplementary material, which is available to authorized users.

## Background

Titanium dioxide (TiO_2_) nanoparticles (NPs), carbon nanotubes (CNTs) and cerium dioxide (CeO_2_) NPs are among the ten most produced NPs worldwide [1]. The production and use of these NPs leads to increasing concentrations in the soil system. Estimated material-flow in sludge treated soils for Europe are 2380 t^−1^ y^−1^ and 0.771 t y^−1^ for TiO_2_ and CNTs, respectively [2]. For CeO_2_ 1400 t y^−1^ are assumed to end up in sludge treated soils worldwide [1]. Thus, all of these three NP types are unintentionally released into the soil ecosystem. One NP type that needs special attention regarding risk assessment in soils is TiO_2_ because these NPs are listed in patents and publications targeted as additives of plant protection products [3, 4]. Thus, if such products were released to the market and applied in the fields, higher concentrations of TiO_2_ NPs would be expected in soils. Due to the potential for increasing amounts of NPs that enter the soil system, it is important to test, whether these NPs affect plants and beneficial soil microorganisms that associate with plant roots and assist plants to acquire nutrients.

Several studies investigated effects of TiO_2_ NPs, CNTs and CeO_2_ NPs on either plants or microorganisms with variable results. For TiO_2_ NPs, contrasting results were found and plant biomass was either decreased or not affected when grown in soil with enhanced TiO_2_ NP concentrations [5–7]. Soil microbial community structures were shown to be altered when treated with TiO_2_ NPs [7–9]. Also CNTs affected plants and soil microbial community structures: the number of flowers and fruits of tomatoes increased, and bacterial community structure changed [10]. In contrast, in another study with much higher CNT concentrations, soil microbial community structure was not affected [11]. Most often, ecotoxicological tests with NPs (TiO_2_, CeO_2_ and CNTs) in soil systems are either performed with plants, or with microorganisms, but the symbiosis of plants and soil microorganisms has rarely been investigated. Plant symbionts provide important ecosystem functions as e.g., nitrogen-fixation by rhizobia in legumes or phosphorus acquisition by arbuscular mycorrhizal fungi (AMF) [12]. One example is red clover which is used for animal feeding and as green manure. Red clover associates with nitrogen-fixing rhizobia bacteria (rhizobia) [13, 14]. Up to 373 kg N ha^−1^ y^−1^ can be fixed by these bacteria in root nodules of red clover plants [15]. Additionally, red clover performs a second symbiosis with AMF [12, 16–18]. These fungi provide plants with soil nutrients, especially immobile nutrients such as phosphorus. Up to 90 % of plant phosphorus is provided by AMF [18]. The two microbial symbionts, AMF and rhizobia, conduct important ecosystem functions [12], and thus it is important to assess whether nitrogen fixation and root colonization by AMF are affected by NPs.

Earlier studies showed that NPs had adverse effect on the legume-rhizobia symbiosis. For soybeans it has been reported that CeO_2_ NPs diminished nitrogen fixation [19], and no effects of TiO_2_ and Fe_3_O_4_ NPs on nodule colonization were found [20]. For barrel clover it has been reported that the number of nodules was decreased and gene expression altered when exposed to biosolids containing Ag, ZnO and TiO_2_ NPs [21, 22]. Peas revealed a delayed nitrogen fixation when exposed to TiO_2_ and ZnO in hydroponic systems [23, 24], and for faba beans, nodulation and nitrogenase activity were delayed by Ag NPs [25]. AMF root colonization has been reported to not being affected in soybeans exposed to TiO_2_ and Fe_3_O_4_ NPs [20], while colonization of white clover roots was increased by Ag and FeO NPs [26]. Because of these effects on legume-rhizobia and AMF systems, it is important to assess whether root colonization by AMF and nitrogen fixation in soil-grown red clover are affected by NPs, e.g. TiO_2_, CeO_2_ and CNTs, because these effects might be species and NP dependent. To our best knowledge, there are no studies available on the effects of CNTs on legume-rhizobia-AMF systems.

In the present study, we investigated the effects of three different NP types, i.e., TiO_2_ NPs, multi-walled CNTs (MWCNTs) and CeO_2_ NPs, on red clover growth, biological nitrogen fixation with rhizobia and on root colonization of AMF in a soil system. We investigated if these NPs affect (1) plant growth, (2) biological nitrogen fixation in plants, (3) AMF root colonization, and (4) phosphorus uptake by red clover. As positive control we chose ZnSO_4_·7H_2_O because Zn^2+^ was reported to decrease plant growth and affect nitrogen fixation of legumes [27]. Effective soil elemental titanium and MWCNT (black carbon) concentrations, their vertical translocation and plant uptake were investigated in detail in a companion paper [28].

## Results

**Fig. 1:**
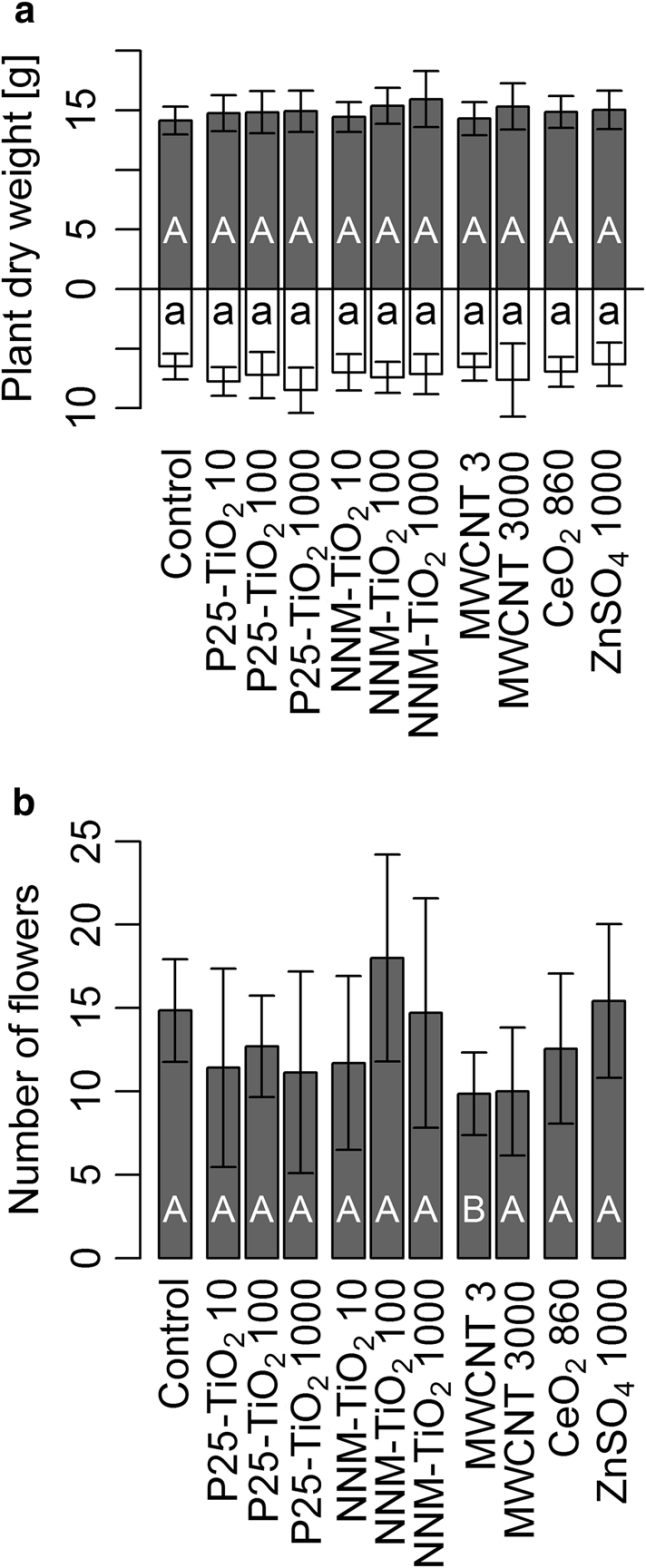
Plant weight and flowers. **a** Red clover plant dry weight divided in shoot (*grey*) and root (*white*), and **b** number of flowers per pot at the end of the 3 month exposure for control, TiO_2_ (P25, non-nanomaterial NNM), MWCNT, CeO_2_ NPs, and ZnSO_4_·7H_2_O. The number behind the treatment name is the nominal concentration in mg kg^−1^. *Error bars* show the standard deviations (n = 7). *Capital letters* show significant differences for shoot biomass and number of flowers, and *small letters* for root biomass compared to the control plants (p ≤ 0.05). The two blocks of starting time were included in the statistical model

Red clover plants were exposed for 14 weeks to agricultural soil spiked with different concentrations of NPs, i.e., TiO_2_ NPs (P25), a bigger non-nanomaterial [29] TiO_2_ particle (NNM-TiO_2_, 20 % particles <100 nm), MWCNTs, CeO_2_ NPs and a ZnSO_4_ treatment. The biomass of red clover plants did not differ between NP spiked substrate and controls without NP addition, both for root and shoot dry weight separately and for total plant dry weight (Fig. 1; Additional file 1: Table S1). Total plant dry weight and effective titanium content per pot were correlated explaining 20 % of variance (Pearson’s correlation: p = 0.041, r = 0.45). The root-shoot ratio was 0.49 ± 0.04 on average, and was also not affected by the presence of NPs (p > 0.05). The number of flowers decreased in the 3 mg MWCNT kg^−1^ soil treatment by 34 % (p = 0.049, Fig. 1; Additional file 1: Table S1). The higher concentration of 3000 mg MWCNT kg^−1^ exhibited a similar decrease in mean number of flowers (33 %), but the variation was higher and therefore the number of flowers was not significantly different from the control plants (p = 0.160).

**Fig. 2:**
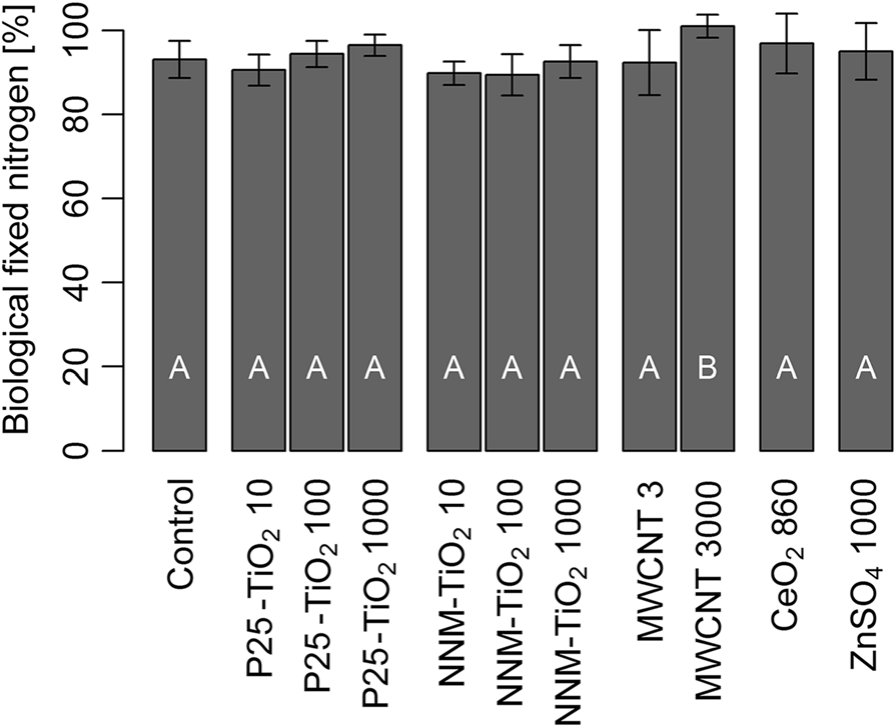
Biological nitrogen fixation. Percentage of atmospheric nitrogen derived from biological nitrogen fixation in red clover shoots for the control, P25 and NNM-TiO_2_, MWCNTs, CeO_2_ NPs, and ZnSO_4_·7H_2_O. The number behind the treatment name is the nominal concentration in mg kg^−1^. Rye grass was used as non-nitrogen fixing plant and the B value was assumed to be zero (see text). *Error bars* show the standard deviations (n = 7). *Capital letters* show significant differences compared to the control plants (p ≤ 0.05)

In addition to plant performance, the interaction of red clover with rhizobia was investigated. All harvested red clover plants contained root nodules and the root nodules had a reddish color which indicates that they fixed nitrogen [14]. In addition, the percentage of fixed nitrogen was assessed based on the ^15^N concentrations of clover and a reference plant (rye grass; see formula 1 in the “Methods” section). The percentages of fixed nitrogen of control red clover plants and NP treated plants were compared, and confirmed that biological nitrogen fixation took place (Fig. 2). All of the treated red clover plants fixed nitrogen and NP application did not affect nitrogen fixation levels in most of the treatments. Only in the 3000 mg MWCNT kg^−1^ treatment, biological nitrogen fixation was increased by 8 % (p = 0.016). Pearson’s correlation revealed a correlation of nitrogen fixation and total biomass of r = 0.28 (p = 0.012).

The second symbiotic partner of red clover, AMF, was assessed by determining root colonization by staining fungal tissue and counting fungal structures by microscopy [30, 31]. In addition the phosphorus content of red clover shoots was assessed, as AMF can contribute significantly to plant P nutrition. Total root colonization by AMF, i.e., % arbuscules, vesicles and hyphae per investigated root intersection, was similar in all treatments (on average 51 ± 4 %; Additional file 1: Figure S1). Also the arbuscular and vesicular colonization revealed no differences between the control and NP treatments (average 23 ± 3 and 6 ± 2 %, respectively; Table 1). Phosphorus concentrations of the red clover shoots were not affected in any of the treatments (Additional file 1: Figure S1b, Table S1). Plant phosphorus content and total root colonization by AMF were not correlated (Pearson correlation coefficient: p = 0.199; r = 0.15).

**Table 1:** Mean values and standard deviation of the arbuscular and vesicular root colonization

	Arbuscular colonization (%)		Vesicular colonization (%)	
	Mean	SD	Mean	SD
Control	23	8	4	2
P25-TiO_2_ 10 mg kg^−1^	22	7	3	3
P25-TiO_2_ 100 mg kg^−1^	22	8	6	9
P25-TiO_2_ 1000 mg kg^−1^	21	10	8	7
NNM-TiO_2_ 10 mg kg^−1^	19	10	7	5
NNM-TiO_2_ 100 mg kg^−1^	24	11	6	3
NNM-TiO_2_ 1000 mg kg^−1^	25	11	7	8
CNT 3 mg kg^−1^	20	9	2	2
CNT 3000 mg kg^−1^	29	7	5	5
CeO_2_ 860 mg kg^−1^	24	9	8	5
ZnSO_4_·7H_2_O 1000 mg kg^−1^	27	8	6	4

## Discussion

In the present study effects of different NPs, i.e., TiO_2_ NPs, MWCNTs and CeO_2_ NPs, on red clover and its symbiosis with rhizobia and AMF were assessed in a soil system. Both tested TiO_2_ treatments (i.e. P25 and NNM-TiO_2_) in all concentrations did not affect plant biomass in our experiment. The absence of effects of TiO_2_ NPs on plant biomass are in agreement with other studies, using different plant species. For example plant growth was not affected when soybeans and corn were exposed to 200 mg TiO_2_ NP kg^−1^ [7] and when tomatoes were exposed to concentrations between 1000 and 5000 mg P25 TiO_2_ NP kg^−1^ [6]. However, in wheat 90 mg TiO_2_ NPs kg^−1^ was shown to decrease plant biomass by 13 % [5]. MWCNTs did not affect red clover biomass in our experiment. Contrary to our findings, MWCNTs have been reported to increase biomass of tomatoes exposed to 50 and 200 µg ml^−1^ MWCNTs per plant [10]. In our experiment red clover biomass did not respond to the CeO_2_ NP treatment, which is in agreement to a study using CeO_2_ NPs at concentrations between 0.1 and 1 g kg^−1^ in an experiment with soybeans [19]. Thus, effects on plant biomass might be influenced by plant species (as shown for the TiO_2_ NPs and MWCNTs) as well as by NP type. All of the above cited studies used different soils. Depending on soil properties, NPs might be differently bound to soil particles [32] which could influence the exposure and the effects of NPs on plants.

The number of flower heads was not affected in both TiO_2_ and CeO_2_ NP treatments at all tested concentrations. However, MWCNTs decreased number of flowers by 34 % (p = 0.049) at the lower concentration (3 mg kg^−1^). The higher MWCNT concentration showed a similar decrease of flower number (33 %), but the variance between the samples was higher and there was no statistically significant difference (p = 0.16). Our results indicate that the number of flowers is sensitive to MWCNTs. Khodakovskaya et al. showed that the number of flower increased significantly, when watered weekly with 50 ml of 50 and 200 µg ml^−1^ MWCNTs per pot for 9 weeks [10]. The direction of the effect was in contrast to our observations. Nevertheless, the number of flowers was affected and further research is needed to determine the mechanism responsible for the effects of MWCNT on flowering.

To test effects of NPs on biological nitrogen fixation, the natural abundance of ^15^N was determined in the red clover shoots and in a reference plant (rye grass) and subsequently the fraction of biological fixed nitrogen in red clover was assessed (see “Methods” section). No nitrogen was added to the pots because increasing the availability of mineral nitrogen has been reported to decline nitrogen fixation rate progressively [33]. The percentage of fixed nitrogen was high and ranged between 89 and 100 % and was not affected by the TiO_2_ NPs in our experiment. These results contrast those of another study performed in a hydroponic system using pea and rhizobia [23]. This study showed that nodulation was negatively affected and that the nitrogen fixation was delayed when TiO_2_ NPs were present. However, it needs to be tested whether the results from hydroponic systems can be directly extrapolated to soil systems. In soils, TiO_2_ NPs interact with soil particles and are probably heteroaggregated with soil particles such as clay minerals [32]. Thus, the plant roots in soils might be less exposed to the NPs than in hydroponic systems and therefore roots and nodules might be less affected in soils, as indicated by the limited transport of TiO_2_ NPs in soils in our experiment [28]. For the higher concentration of MWCNTs (3000 mg kg^−1^), nitrogen fixation increased by 8 % (p = 0.01) compared to the control and 100 % of the nitrogen content in the shoots originated from nitrogen fixation. Even though the biomass and total nitrogen content of these MWCNT treated plants were not different from those in the control treatment, correlation between biologically fixed nitrogen and total biomass over all treatments was significant but only 8 % of the variation could be explained (R^2^ = 0.08; p = 0.012). This indicates that enhanced nitrogen fixation had only a small beneficial effect on plant growth. In our experiment, nitrogen fixation was not affected by CeO_2_ NPs. For soybeans however, the CeO_2_ NPs have been reported to decrease nitrogen fixation potential up to 80 % [19]. This reference investigated a different plant species and effects of NPs might be plant and rhizobia species specific [19]. Also the use of different soils with different soil characteristics might influence the results. Further experiments are needed to consolidate our understanding of the mechanisms of how NPs affect nitrogen fixation.

Total arbuscular, as well as vesicular root colonization of red clover by AMF were not affected in any of the treatments. In support of this finding, but again with another plant species, Burke et al. [20] reported no effects of TiO_2_ NPs on AMF root colonization in soybeans using a DNA based approach instead of counting the root colonization. AMF provide plants with nutrient, such as phosphorus [17, 34]. Therefore we assessed phosphorus content in red clover shoots at the harvest. Phosphorus content of red clover shoots was not affected in any of the treatments and there was no correlation between plant phosphorus content and total AMF root colonization (p = 0.2). Again, for TiO_2_ NPs this is in agreement with Burke et al. who did not find differences in phosphorus content of soybean leaves [20]. Even though root colonization was not affected by the tested NPs in our experiments, community structure of AMFs in soils might change as shown in Burke et al. [7].

Contrary to our expectations, the ZnSO_4_ control did not affect any of the measured endpoints. It is known that Zn^2+^ availability is limited at high soil pH conditions [35]. Soil pH was 7.7 [28] and the concentration added was probably not high enough to release enough free Zn^2+^ to cause harmful effects.

The amount of NPs applied to the soil was high and partly outside the exposure range expected in the field. They were chosen to represent a potential agricultural application scenario, where fluxes between several micrograms to grams of NPs per kilogram of soil are estimated [3]. The highest concentration also simulates accidental spill during transport or pollution in industrial areas or in the field. In our experiment also lower concentrations, i.e. 10 and 100 mg kg^−1^ soil, were tested. This approach ensures that potential negative effects can be detected before a NP is widely used and applied. This approach also facilitates the detection of potential harmful NPs in comparison to non-toxic or less harmful NP. Moreover, in order to be able to detect and measure concentrations of some NPs in the environment (e.g. titanium oxides for this study), high amounts have to be added because element like titanium occur naturally in the soil and the concentrations added need to be higher as natural background levels. For instance, for TiO_2_ NPs the lowest concentration of 10 mg kg^−1^ is realistic in comparison with estimations for soils treated with NP containing plant protection products, while the highest tested concentration (1000 mg kg^−1^) rather represents a worst case scenario [3]. For MWCNTs, yearly increases of estimated environmental concentrations are estimated to range from 5 to 990 ng kg y^−1^ [2]. Hence, both tested concentrations in our experiment are above natural values and represent an upper limit. The addition of these high concentrations was necessary to distinguish the added MWCNTs from the black carbon background of the soil [28, 36]. New methods are currently being developed to distinguish NPs from natural backgrounds as reviewed by others [37, 38]. Further research is needed to measure and characterize NPs in soils at predicted environmental concentrations, both for fate and behavior studies, and to accompany environmentally relevant ecotoxicological tests.

## Conclusions

The investigated TiO_2_ NPs and CeO_2_ NPs did not affect red clover growth, biological nitrogen fixation and AMF root colonization. Opposite to other studies with TiO_2_ and CeO_2_ that observed effects on N fixing legumes, no effects were observed here with red clover. Further research is needed to search for general patterns and investigate the mechanisms behind such effects. MWCNTs increased nitrogen fixation and decreased the number of flowers compared to the control treatment, which might affect fitness of red clover. However, these effects occurred at concentrations much higher than expected in the environment.

## Methods

### NPs used for the experiment

P25 (Sigma Aldrich, USA, art. No. 718467) with a particle size of 29 ± 9 nm [28] was used as representative for TiO_2_ NPs. In addition, NNM-TiO_2_ (Sigma Aldrich, USA, Art. No. 232033) with an average particle size of 145 ± 46 nm [28] was used as non-nano-material, i.e. less than 50 % NPs [29]. MWCNTs were purchased from Cheap Tubes Inc. (USA). They had a length of 10–30 μm, outer diameter of 20–30 nm, a purity of >95 % and an elemental carbon content of >98 % (Additional file 1: Table S2) [28]. CeO_2_ NPs (Sigma Aldrich, USA, art. No. 700290) had a diameter of less than 50 nm with cubic crystal structure according to the manufacturer’s specifications.

### Mixing NPs into the soil

For preparing the substrate, soil classified as brown earth with a sandy loamy to loamy fine fraction was collected from an agricultural field at Agroscope Institute for Sustainability Sciences in Zurich, Switzerland (coordinates N47° 25′ 39.564″ E8° 31′ 20.04″). For this, the top 5 cm were removed and the underlying 15 cm soil were collected and sieved (<0.5 cm). The soil was mixed with quartz sand (50 % v/v) and then characterized as described by Gogos et al. (Additional file 1: Table S3) [28]. Nutrient contents in the mixture were 37.6 mg kg^−1^ phosphorus and 85.3 mg kg^−1^ potassium determined by ammonium acetate EDTA extraction [39]. Soil pH was 7.7. Each of the different NPs was premixed in 300 g substrate (soil and sand) on an overhead mixer (Turbula T2F, Switzerland) in 500 ml Schott bottles by adding 0.3, 3 and 30 g of P25 or NNM-TiO_2_, 90 mg and 88 g MWCNTs, 25 g CeO_2_ NPs and 30 g ZnSO_4_·7H_2_O (Sigma Aldrich, USA, art. No. Z0251), respectively. P25 (30 g) and MWCNTs (88 g) revealed a too large volume for the 500 ml Schott bottles, necessitating the division of the soil and additives into several bottles (300 g of substrate for each bottle). For P25 15 g were added to two Schott bottles, and for MWCNTs 22 g were added to four bottles. Each of these pre-mixtures was diluted with substrate to a total volume of 30 kg and mixed in a cement mixer for 6 h.

### Experimental setup

**Fig. 3:**
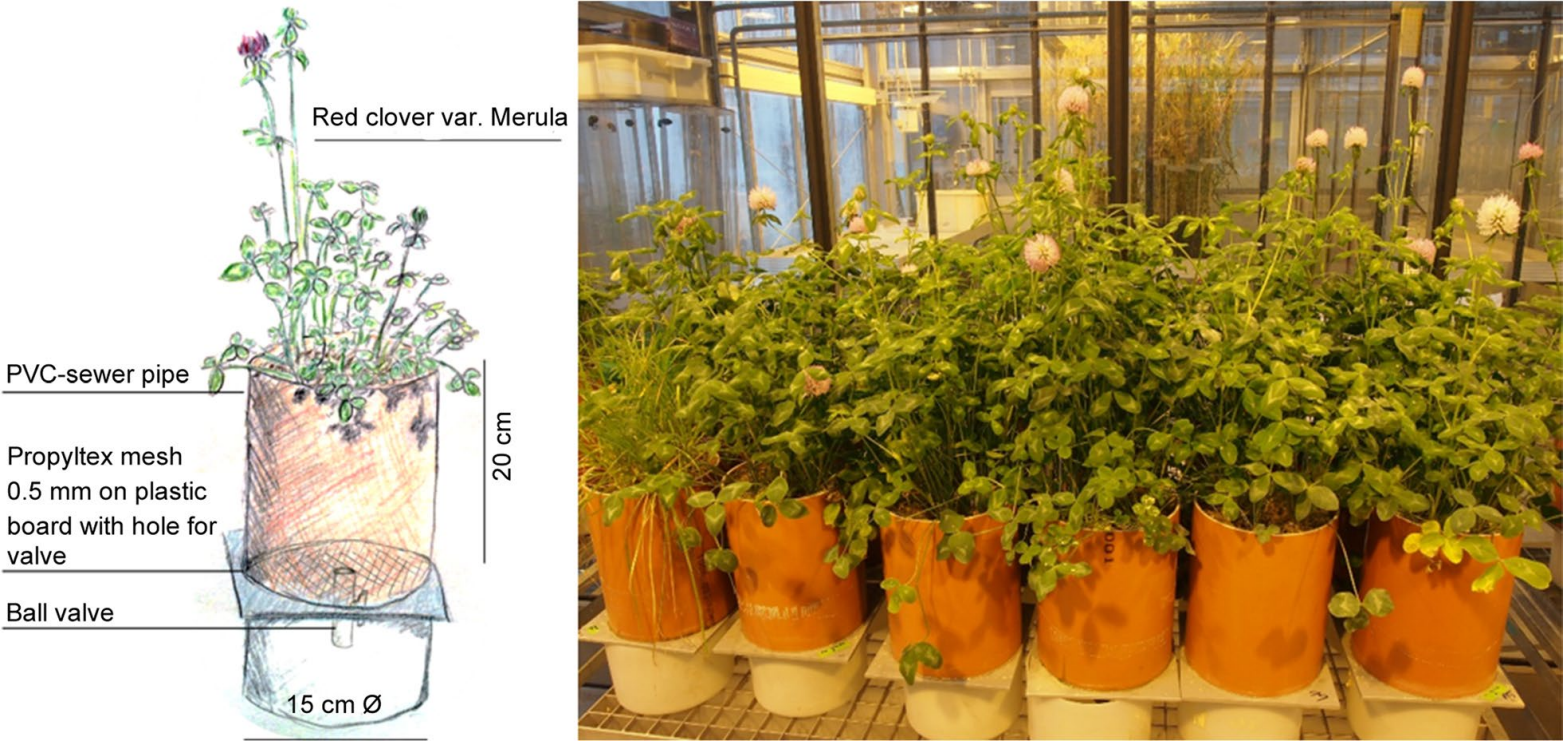
Experimental setup. Sketch of the experimental setup of the pots and picture of a part of the pots in the greenhouse 12 weeks after the start of the experiment. All of the pots were randomly arranged in the greenhouse

Pots were prepared by gluing PVC-sewer pipes (15 cm diameter, 20 cm long) on a plastic board with a ball valve as draining device (Fig. 3). A plastic mesh (Propyltex 500 µm, Sefar, Switzerland) was placed on the top of the valve to prevent blockage of the valve by the substrate. Pots were filled with a 500 g quartz sand layer as drainage and 3.3 kg spiked substrate or control substrate. Seven replications per treatment were prepared, i.e., control, P25, NNM-TiO_2_, MWCNT, CeO_2_ NPs, and ZnSO_4_·7H_2_O. Total elemental titanium, black carbon (BC, for MWCNT treatments) and elemental cerium concentrations were determined in the substrate as described in the accompanying study [28]. Average total elemental titanium concentration of the highest tested concentrations was determined at the end of the experiment using X-ray fluorescence (XRF) and was 1332 ± 100 for the control treatment without titanium, 2059 ± 105 for 1000 mg kg^−1^ (nominal) P25 and 2007 ± 79 mg kg^-1^ for the NNM-TiO_2_ treated soils, respectively [28]. For MWCNT the background of BC in control soils was on average 0.50 ± 0.06 mg g^−1^ and BC concentration in MWCNT 3000 mg kg^−1^ treated soil was 2400 ± 100 mg kg^−1^ as quantified by chemothermal oxidation [28]. Average elemental cerium concentration in the 830 mg kg^−1^ CeO_2_ treatment was 416 ± 19 mg kg^−1^ determined with XRF at the end of the experiment.

### Cultivation of red clover in NP spiked substrate

Red clover (*Trifolium pratense* var. Merula) was germinated on filter paper for 5 days. Thereafter, seven seedlings of equal size were transferred to the pots with substrate spiked with NPs or control soils in a greenhouse (16 h 25 °C 300 W m^2^, and 8 h 16 °C in the dark). In addition seven pots with ryegrass (*Lolium perenne* var. Arolus) were prepared in the same way. These plants were grown because a non-nitrogen-fixing plant was needed to estimate biological fixed nitrogen in red clover (see below). The experiment was started in two blocks (n = 4 and 3, respectively), time-shifted with 1 week difference. All pots were regularly watered to keep the water holding capacity between 60 and 70 % (controlled by weighing and adding every time the same amount of water to all of the pots). Clover was fertilized after 6 and 9 weeks with 10 ml of · KH_2_PO_4_ (5 mM), MgSO_4_·7H_2_O (1 mM), KCl (50 µM), H_3_BO_3_ (25 µM), MnSO_4_·H_2_O (1.3 µM), ZnSO_4_·7H_2_O (2 µM), CuSO_4_·5H_2_O (0.5 µM), (NH_4_)6Mo_7_O_27_·4H_2_O (0.5 µM), and Fe(III) EDTA (20 µM). This is comparable to a phosphorus addition of 1.7 kg P ha^−1^.

After 14 weeks NP exposure of red clover, the number of flowers (flower heads) was determined and the plant shoots were harvested. Soil cores were taken to assess NP concentration as described in Gogos et al. [28]. Roots were separated from the soil and washed. Then the roots were cut in 1 cm pieces, mixed in water and a randomized root subsample of approximately 2 g was taken for determining the AMF colonization. Roots were padded with a paper towel and weighed. The subsample was weighed separately and then stored at 4 °C in 50 % ethanol in Falcon tubes until the colonization was determined. The remaining roots as well as the red clover and ryegrass shoots were dried at 70 °C until they reached constant dry weight and dry weight of roots, shoots and total biomass (root + shoot weight) were determined. The dry weight of the AMF colonization root sample was calculated using the dry/wet weight ratio of the root sample. This AMF sample dry weight was added to the total root dry weight. Shoots of red clover and ryegrass were ground with a centrifugation mill (0.2 mm sieve, Retsch ZM200, Germany) and 2 mg samples were sent for ^15^N analysis by isotope ratio mass spectrometry at the stable isotope facility at Saskatchewan University (Canada). Root colonization of AMF was analyzed by microscopy following the protocols of Vierheilig et al. [31] for staining the roots and McGonigle et al. [30] for counting the AMF structures. In short, roots were rinsed with deionized water, and transferred to 10 ml 10 % KOH for 20 min at 80 °C. Roots were rinsed again with water and stained in 5 % (v/v) ink (Parker Quink, black) in vinegar for 15 min at 80 °C. After rinsing the stained roots, they were transferred to 50 % glycerol for storage until root colonization was assessed. For microscopy, the root pieces were aligned in parallel onto a glass slide, covered with 50 % glycerol, and the roots were covered with a cover slip [30]. AMF structures in plant roots, i.e., hyphae, arbuscules, and vesicles, were counted for 100 intersections as described by McGonigle et al. [30]. Phosphorus content of shoots was assessed by ICP-OES using a hydrochloric acid digestion of the ashed residues [40].

Nitrogen fixation [%] was calculated using Eq. 1 where B is the value of δ^15^N of shoots of plants, that are fully dependent upon nitrogen fixation [33]. For our experiment, a B value of 0 was assumed which reflects δ^15^N of plants that are totally dependent on nitrogen fixation. The reference plant δ^15^N was derived from the ryegrass shoots.

1 $$\begin{aligned} {\% } {\text{ Nitrogen fixation}} &= \frac{{\delta^{15} {\text{N of reference plant }} - \delta^{15} {\text{N of N}}_{2} {\text{ fixing plant}}}}{{\delta^{15} {\text{N of reference plant}} - {\text{B}}}} \quad\times \frac{100}{1} \end{aligned}$$

### Statistics

All statistical analyses were performed with R [41]. A generalized linear model with Gaussian distribution was applied to determine differences of each treatment to the control. Thereby the two blocks of the different starting dates of the pot experiment were included as error term. The model was analyzed for homogeneity (Bartlett test) and normality (Shapiro test). Additionally a Dunnett test was performed (R library SimComp) using adjusted p-values for multiple testing [42] when normality and homogeneity were fulfilled. For non-normal residuals or inhomogeneous data, a Mann–Whitney test was used and p-values were adjusted for multiple testing according to Benjamini and Hochberg [43]. Pearson’s correlations were calculated with the R command cor.test.

## Additional file

10.1186/s12951-016-0188-7Supplementary information on arbuscular mycorrhizal fungal root colonization, multi walled carbon nanotubes (MWCNT) characterization, and soil properties.

## Abbreviations

AMF: arbuscular mycorrhizal fungi
CeO_2_: cerium dioxide
CNT: carbon nanotubes
MWCNT: multiwalled carbon nanotubes
^15^N: nitrogen isotope
NNM-TiO_2_: non-nanomaterial titanium dioxide
NP: nanoparticle
TiO_2_: titanium dioxide
