# Supplementary material for: Effect of nanoparticles on red clover and its symbiotic microorganisms
Source: J Nanobiotechnology. 2016 May 10;14:36. doi: 10.1186/s12951-016-0188-7 (PMC4862186; doi:10.1186/s12951-016-0188-7)
Supplement: Supplementary file 1 — 10.1186/s12951-016-0188-7Supplementary information on arbuscular mycorrhizal fungal root colonization, multi walled carbon nanotubes (MWCNT) characterization, and soil properties. [file 12951_2016_188_MOESM1_ESM.docx]

# Supplementary Information

# Effect of nanoparticles on red clover and its symbiotic microorganisms

Janine Moll^a^, Alexander Gogos^a^, Thomas D. Bucheli^a^, Franco Widmer^a^, Marcel G.A. van der Heijden^a^*

^a^Agroscope, Institute for Sustainability Sciences ISS, 8046 Zurich, Switzerland

mollj@bluewin.ch, alexander.gogos@eawag.ch, franco.widmer@agroscope.admin.ch, thomas.bucheli@agroscope.admin.ch

*Corresponding author. Tel.: +41 58 468 72 78; fax: +41 58 468 72 01; e-mail: marcel.vanderheijden@agroscope.admin.ch

**
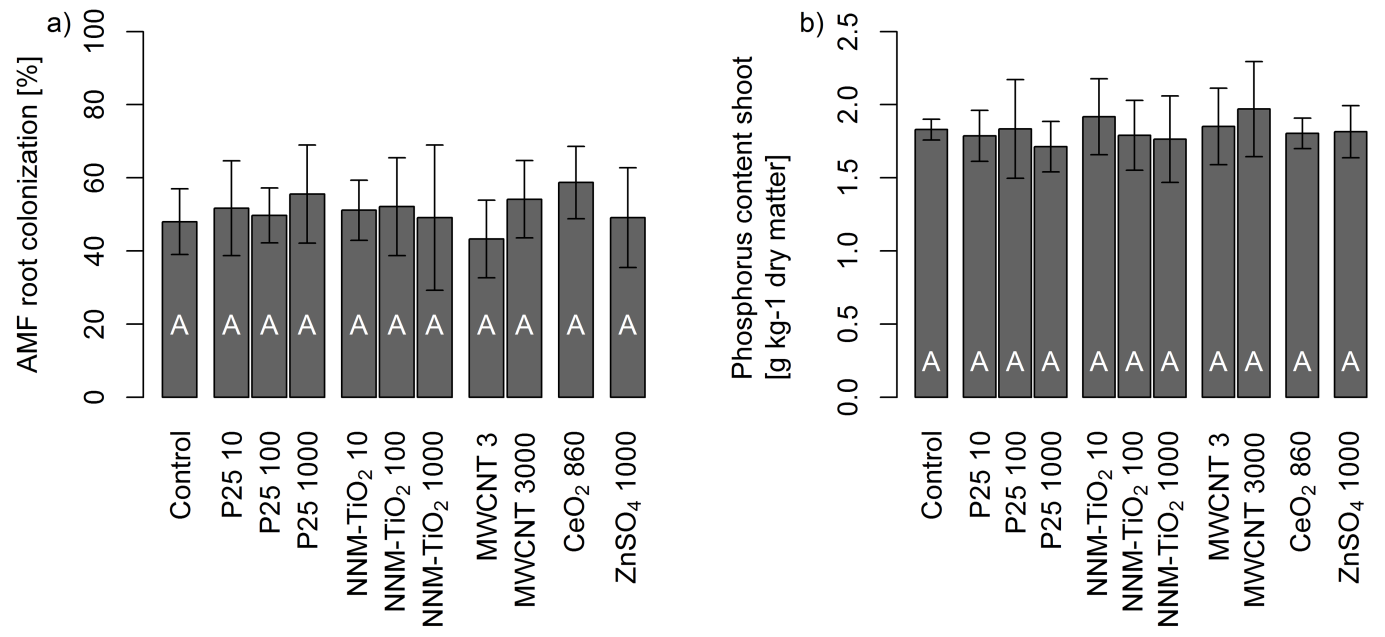
**

**Figure S1:** **Arbuscular mycorrhizal root colonization and phosphorus content.** a) Total root colonization (percentage of arbuscules, vesicles and hyphae per assessed root intersection) of red clover roots and b) phosphorus concentration in red clover shoots for the control, P25 and NNM-TiO_2_, CNTs, CeO_2_ NPs, and ZnSO_4_^.^7H_2_O. The numbers after the treatment name indicate the nominal concentrations in mg kg^-1^. Error bars show the standard deviations (n=7). Different letters indicate statistically significant differences between the treatments (p≤0.05).

**Table S1: Summaries of the statistical tests applied.** A generalized linear model (glm) was applied to test the model assumptions and to check if the two blocks of the starting times of the experiment were significant different from each other. If the residuals were normal and homogeneous, a Dunnett’s test was performed and p values were adjusted for multiple comparisons. For not normal residuals, a Mann-Whitney test was applied. Statistical significant p values (p<0.05) are indicated in bold.

|  | shoot dry weight [g] | | | | |  | root dry weight [g] | | | | |  | root shoot ratio | | | | |  | number of flowers | | | | |  | biological nitrogen fixation | |
| --- | --- | --- | --- | --- | --- | --- | --- | --- | --- | --- | --- | --- | --- | --- | --- | --- | --- | --- | --- | --- | --- | --- | --- | --- | --- | --- |
|  | glm | |  | Dunnett | |  | glm | |  | Dunnett | |  | glm | |  | Dunnett | |  | glm | |  | Dunnett | |  | Mann-Whitney | |
|  | t | p _adj_ |  | t | p _adj_ |  | t | p _adj_ |  | t | p _adj_ |  | t | p _adj_ |  | t | p _adj_ |  | t | p _adj_ |  | t | p _adj_ |  | W | p _adj_ |
| Block | 6.4 | **<0.001** |  |  |  |  | -0.9 | 0.730 |  |  |  |  | -3.3 | **0.008** |  |  |  |  | 2.1 | 0.186 |  |  |  |  |  |  |
| P25 10 mg kg^-1^ | 0.9 | 0.453 |  | 0.8 | 0.977 |  | 1.4 | 0.681 |  | 2.1 | 0.354 |  | 1.1 | 0.872 |  | 1.7 | 0.563 |  | -1.3 | 0.335 |  | -1.4 | 0.781 |  | 34 | 0.437 |
| P25 100 mg kg^-1^ | 1.0 | 0.419 |  | 0.9 | 0.971 |  | 0.8 | 0.730 |  | 0.9 | 0.975 |  | 0.5 | 0.918 |  | 0.5 | 1.000 |  | -0.8 | 0.486 |  | -1.3 | 0.808 |  | 19 | 0.654 |
| P25 1000 mg kg^-1^ | 1.1 | 0.419 |  | 1.0 | 0.943 |  | 2.2 | 0.208 |  | 2.4 | 0.237 |  | 2.0 | 0.183 |  | 1.5 | 0.668 |  | -1.5 | 0.335 |  | -1.4 | 0.726 |  | 11 | 0.357 |
| NNM-TiO_2_ 10 mg kg^-1^ | 0.4 | 0.724 |  | 0.5 | 1.000 |  | 0.5 | 0.759 |  | 0.7 | 0.994 |  | 0.5 | 0.918 |  | 0.5 | 1.000 |  | -1.2 | 0.335 |  | -1.4 | 0.771 |  | 33 | 0.437 |
| NNM-TiO_2_ 100 mg kg^-1^ | 1.8 | 0.217 |  | 1.7 | 0.548 |  | 1.0 | 0.730 |  | 1.4 | 0.728 |  | 0.4 | 0.918 |  | 0.5 | 0.999 |  | 1.2 | 0.335 |  | 1.2 | 0.859 |  | 34 | 0.437 |
| NNM-TiO_2_ 1000 mg kg^-1^ | 2.6 | **0.044** |  | 1.8 | 0.501 |  | 0.7 | 0.730 |  | 0.8 | 0.977 |  | -0.2 | 0.976 |  | -0.3 | 1.000 |  | -0.1 | 0.956 |  | -0.1 | 1.000 |  | 25 | 1.000 |
| CNT 3 mg kg^-1^ | 0.2 | 0.807 |  | 0.2 | 1.000 |  | 0.0 | 0.965 |  | 0.1 | 1.000 |  | 0.0 | 0.981 |  | 0.0 | 1.000 |  | -2.0 | 0.186 |  | -3.3 | **0.049** |  | 23 | 0.992 |
| CNT 3000 mg kg^-1^ | 1.7 | 0.217 |  | 1.4 | 0.754 |  | 1.2 | 0.681 |  | 0.9 | 0.960 |  | 0.5 | 0.918 |  | 0.4 | 1.000 |  | -1.9 | 0.186 |  | -2.6 | 0.160 |  | 2 | **0.013** |
| CeO_2_ 860 mg kg^-1^ | 1.0 | 0.419 |  | 1.1 | 0.919 |  | 0.5 | 0.759 |  | 0.7 | 0.993 |  | 0.1 | 0.981 |  | 0.2 | 1.000 |  | -0.9 | 0.486 |  | -1.1 | 0.904 |  | 15 | 0.437 |
| ZnSO_4_^.^7H_2_O 1000 mg kg^-1^ | 1.3 | 0.397 |  | 1.2 | 0.864 |  | -0.2 | 0.924 |  | -0.2 | 1.000 |  | -0.8 | 0.918 |  | -0.9 | 0.955 |  | 0.2 | 0.899 |  | 0.3 | 1.000 |  | 16 | 0.437 |
| Ryegrass control |  |  |  |  |  |  |  |  |  |  |  |  |  |  |  |  |  |  |  |  |  |  |  |  | 49 | **0.006** |
|  | %nitrogen in shoots | | | | |  | AMF total colonization | | | | |  | Ratio Ndfa / total biomass | | | | |  | Phosphorus [g kg^-1^ dry matter] | | | | |  |  |  |
|  | glm | |  | Dunnett | |  | glm | |  | Dunnett | |  | glm | |  | Dunnett | |  | glm | |  | Dunnett | |  |  |  |
|  | t | p _adj_ |  | t | p _adj_ |  | t | p _adj_ |  | t | p _adj_ |  | t | p _adj_ |  | t | p _adj_ |  | t | p _adj_ |  | t | p _adj_ |  |  |  |
| Block | -5.9 | **<0.001** |  |  |  |  | -4.0 | **0.001** |  |  |  |  | -2.6 | 0.120 |  |  |  |  | -3.7 | 0.003 |  |  |  |  |  |  |
| P25 10 mg kg^-1^ | 0.9 | 0.716 |  | 0.9 | 0.967 |  | 0.6 | 0.793 |  | 0.6 | 0.996 |  | -1.9 | 0.220 |  | -2.5 | 0.193 |  | -0.3 | 0.944 |  | -0.6 | 0.999 |  |  |  |
| P25 100 mg kg^-1^ | -1.9 | 0.850 |  | -0.1 | 1.000 |  | 0.3 | 0.846 |  | 0.4 | 1.000 |  | -0.8 | 0.701 |  | -0.8 | 0.978 |  | 0.1 | 0.944 |  | 0.0 | 1.000 |  |  |  |
| P25 1000 mg kg^-1^ | -0.4 | 0.843 |  | -0.4 | 1.000 |  | 1.3 | 0.607 |  | 1.2 | 0.824 |  | -1.6 | 0.266 |  | -2.6 | 0.172 |  | -0.9 | 0.944 |  | -1.6 | 0.670 |  |  |  |
| NNM-TiO_2_ 10 mg kg^-1^ | 1.1 | 0.696 |  | 1.1 | 0.882 |  | 0.5 | 0.793 |  | 0.7 | 0.993 |  | -1.3 | 0.390 |  | -1.7 | 0.593 |  | 0.7 | 0.944 |  | 0.8 | 0.982 |  |  |  |
| NNM-TiO_2_ 100 mg kg^-1^ | -0.3 | 0.850 |  | -0.2 | 1.000 |  | 0.7 | 0.793 |  | 0.7 | 0.993 |  | -2.4 | 0.120 |  | -3.3 | 0.051 |  | -0.3 | 0.944 |  | -0.4 | 1.000 |  |  |  |
| NNM-TiO_2_ 1000 mg kg^-1^ | 0.9 | 0.716 |  | 0.6 | 0.995 |  | 0.2 | 0.846 |  | 0.1 | 1.000 |  | -1.8 | 0.220 |  | -1.8 | 0.537 |  | -0.5 | 0.944 |  | -0.6 | 0.999 |  |  |  |
| CNT 3 mg kg^-1^ | 0.6 | 0.799 |  | 0.6 | 0.999 |  | -0.8 | 0.793 |  | -0.9 | 0.962 |  | -0.3 | 0.977 |  | -0.4 | 1.000 |  | 0.3 | 0.944 |  | 0.2 | 1.000 |  |  |  |
| CNT 3000 mg kg^-1^ | 0.7 | 0.759 |  | 0.6 | 0.998 |  | 1.0 | 0.720 |  | 1.2 | 0.862 |  | 0.0 | 0.977 |  | 0.0 | 1.000 |  | 1.2 | 0.912 |  | 1.0 | 0.938 |  |  |  |
| CeO_2_ 860 mg kg^-1^ | 0.4 | 0.843 |  | 0.6 | 0.998 |  | 1.8 | 0.292 |  | 2.1 | 0.317 |  | -0.2 | 0.977 |  | -0.2 | 1.000 |  | -0.2 | 0.944 |  | -0.5 | 1.000 |  |  |  |
| ZnSO_4_^.^7H_2_O 1000 mg kg^-1^ | 1.8 | 0.236 |  | 1.4 | 0.709 |  | 0.2 | 0.846 |  | 0.2 | 1.000 |  | -0.1 | 0.977 |  | -0.2 | 1.000 |  | -0.1 | 0.944 |  | -0.2 | 1.000 |  |  |  |
| Ryegrass control | -21.8 | **<0.001** |  | -27.9 | **<0.001** |  |  |  |  |  |  |  |  |  |  |  |  |  |  |  |  |  |  |  |  |  |

**Table S2: Characteristics of the MWCNTs.**

|  | | **Value** |
| --- | --- | --- |
| **Nominal TOC^1^ g/kg dw** | | 983.5 |
| **Length^1^ (µm)** | | 10-30 |
| **Od^1^ (nm)** | | 20-30 |
| **Id^1^ (nm)** | | 5-10 |
| **Functionalization^1^ (wt%)** | | pristine |
| **Elemental content (Quantified by energy dispersive X-ray spectroscopy EDX)** | **C^1^ (wt%)** | 98.35 |
|  | **Cl^1^ (wt%)** | 0.45 |
|  | **Fe^1^ (wt%)** | 0.26 |
|  | **Ni^1^ (wt%)** | 0.94 |
| **Purity^1^ (wt%)** | | >95 |
| **Ash**^1^ **wt%** | | <1.5% |
| **Specific surface area (BET)^1^ (m^2^/g)** | | >110 |
| **Electrical conductivity^1^ (S/cm)** | | >100 |
| **TOC**^2^ **(g/kg_CNT_)** | | 977 ± 24 |
| **BC 375˚C**^2^ **(g/kg_CNT_)** | | 658 ± 28 |
| **C after CTO-375**^2^ **(wt%)** | **Average** | 67.36 |
|  | **Stdev** | 28 |
| **Cd^2,3^ (µg/kg )** | | <285 |
| **Co^2,3^ (mg/kg)** | | 11329.78 |
| **Cr^2,3^ (mg/kg)** | | 17.14 |
| **Cu^2,3^ (mg/kg)** | | 3.8 |
| **Fe^2,3^ (mg/kg)** | | 2629.17 |
| **Ni^2,3^ (mg/kg)** | | 927.17 |
| **Pb^2,3^ (mg/kg)** | | 1.03 |
| **Zn^2,3^ (mg/kg)** | | <7.5 |
| **Mn^2,3^ (mg/kg)** | | 1.6 |
| **Mg^2,3^ (mg/kg)** | | 30.2 |
| **Al^2,3^ (mg/kg)** | | 14.7 |
| **Ca^2,3^ (mg/kg)** | | 258.6 |
| **Sum (mg/kg)** | | 15213.1 |
| **Sum g/kg** | | 15.2 |
| **Metals (%)** | | 1.5 |

^1^Data provided by the manufacturer

^2^Own measurements

^3^Determined using microwave acid digestion and ICP-MS

**Table S3:** Characterization of the soil substrate used in the experiments analyzed by the companion study of Gogos et al. [1].

| **Parameter** | **Value** | **SD** |
| --- | --- | --- |
| Org. C | 0.55 | 0.03 |
| CEC mmol+/kg | 6 |  |
| CaCO_3_ % | 2.6 |  |
| pH | 7.7 |  |
| max. WHC g H_2_O/g dry soil | 0.308 |  |
| Sand/silt/clay % | 86.1/6.3/6.7 | 0/0/0.5 |

## References

1. Gogos A, Moll J, Klingenfuss F, van der Heijden M, Irin F, Green M, Zenobi R, Bucheli T: **Vertical transport and plant uptake of nanoparticles in a soil mesocosm experiment.** Submitted to Journal of Nanobiotechnology.
